# Delivering forestry courses online: experiences, lessons learned, and future of forestry online education in the Asia Pacific

**DOI:** 10.1007/s11676-022-01555-5

**Published:** 2022-11-07

**Authors:** Anil Shrestha, Jodi Crawford, Hailan Chen, Shiyi Zhang, Na Zhong, Michelle Zeng, Guangyu Wang

**Affiliations:** 1 Asia Forest Research Center, Faculty of Forestry, University of British Columbia, Vancouver, BC V6T 1Z4 Canada; 2 Center of Teaching, Learning, and Technology, University of British Columbia, Vancouver, BC V6T 1Z4 Canada; 3 Asia-Pacific Network for Sustainable Forest Management and Rehabilitation, Beijing, 100102 People’s Republic of China

**Keywords:** Sustainable Forest Management, Online forestry education, Pedagogy innovation, Covid-19, Lessons learned, Asia Pacific

## Abstract

Innovation in forestry education is needed to address changing contexts of the positionality of forests. This is particularly significant in the Asia–Pacific region, where deforestation and degradation are high. However, the accessibility of high-quality forestry education to address changing regional and global contexts is lacking. A series of innovative sustainable forest management (SFM) open education resource (OER) courses were developed and implemented to improve the accessibility of SFM education to enhance teaching quality, curriculum, and research capacity of universities in the Asia-Pacific Region. To evaluate the SFM-OER program in terms of student experiences, this study investigated student achievement, perceived success of the pedagogical approach and instructional design, and perceived effectiveness of the learning activities in promoting active and transformative learning through the assessment of a 1,191-course feedback survey between 2018 and 2020, including the global pandemic. This study revealed that the program attracted diverse student demographics, including a higher proportion of female students majoring in forestry, ecology, and other environmental studies. Their primary motivation to participate in the courses was to gain international experience, followed by the flexibility of online learning, mandatory course requirements, and earning course credits. Students were satisfied with the Canvas learning management system. Most students spent less than 5 to 10 h of their weekly time in the course and agreed or strongly agreed that the workloads were manageable. Students reflected positively on various learning activities and assignments, such as watching lecture videos, taking quizzes, reading and summarizing, having discussions, and peer review writing. However, they did not clearly prefer specific learning activities, signifying the importance of using diverse learning activities to satisfy diverse individual learning styles in online settings. This analysis contributes to the further development of student-centered pedagogical development for online learning and provides insight into the ways forward for online higher forestry education, while repurposing existing OER courses in a post-Covid-19 era.

## Background of forestry and online forestry education

### Forestry education and online learning

Innovation in forestry education is needed to address changing contexts of the forests due to climate change, biodiversity conservation, forest restoration, poverty reduction, the engagement of local and indigenous peoples, and global corporate behavior. Thus, forestry education programs will need to adopt an interdisciplinary approach to build capacity of future graduates with a wide breadth of knowledge and skillsets who can collaborate across geographic and political boundaries to understand and address the many complexities and interrelated phenomena involved in managing forests in these ever-changing environmental, technological, sociological, political, and economic atmospheres (Innes 2005; Andreade et al. 2014). Achieving this will be a central challenge for those involved in designing forestry education programs.

Online forestry education can serve as an effective tool by providing cutting-edge alternate delivery methods to complement traditional classroom instruction to reach a larger audience for developing key competencies of employees and future employees in the forestry sector using various forms of training and academic degrees (Standiford 2015; Jegatheswaran et al. 2018; Mushkarova et al. 2020). The Covid-19 pandemic provided an unexpected and widespread opportunity for online forestry education to develop further and explore. This crisis facilitated forestry instructors to diversify their pedagogical approaches, digital skill sets, and resources to promote interactive learning between faculty and students through remote methods to achieve higher-order learning outcomes in online settings. In this changing context, exploring how online pedagogy can be applied to higher forestry education and how it can be improved as a tool to augment and enhance traditional learning is anticipated to develop further (Mushkarova et al. 2020; Pastukh and Zhuk 2021). This is particularly relevant in the Asia–Pacific region, where unprecedented rates of forest loss are occurring as well as the advancement of forest management technologies, expanding economies, climate change, and conflicts in forest governance (FAO 2019) and a lack of access to high-quality forestry courses relevant to the changing global context (AP-FECM 2018). This makes the region a particularly important area of interest for improved forestry education — where enrollment in forestry programs is expanding rather than shrinking, (except for The Philippines and Indonesia) (Ratnasingam et al. 2013). As a result, there is ample opportunity and need to develop strong forestry curriculums within this region that will help build capacity of well-educated foresters who can sustainably manage forests into the future (de Jong et al. 2021; Saengcharncha et al. 2021).

### Innovation and application of SFM online forestry education

In this background, a suit of 14 innovative, sustainable forest management open education resource (SFM-OER) courses was developed with funding from the Asia–Pacific Network for Sustainable Forest Management and Rehabilitation (APFNet) to address the lack of access to high-quality forestry courses and the changing context of forestry. Several instructors led online courses — repurposing SFM-OER courses have been offered since 2016 as open enrollment free of charge, targeting graduate and undergraduate students, instructors, policymakers, and mid-career professionals in Asia Pacific regions through the Asia–Pacific Forest Education Mechanism (AP-FECM) executive office, a regional platform on forestry education cooperation and exchange based in the Faculty of Forestry at the University of British Columbia (UBC). The COVID-19 pandemic resulted in an unexpected opportunity for the application of these repurposed OER courses when face-to-face learning was almost not possible in universities. During this critical time, the SFM-OER courses were readily adapted, and two sessions were offered in February 2020 (7 courses) and September 2020 (5 courses). These online teaching courses reached ~ 2700 students representing various universities in the Asia–Pacific and South America.

###  Justification of the current research objectives

As the first program of its kind, the innovative learning format of the SFM-OER program is a step forward in online higher forestry education. It maintains the high-quality and innovative pedagogical characteristics of the program and helps the courses remain up to date with changing trends in forestry and related fields. The flexibility and accessibility of online forestry education can meet the growing needs of forestry education by offering easy access to many courses with which learners can build broad and interdisciplinary knowledge foundations and create networking opportunities for foresters worldwide. However, these benefits can only be obtained if the pedagogical design of these online courses is capable of fostering learning outcomes that are attractive and effective to students for them to want to participate. The sector of online higher forestry education is a relatively new field of pedagogy, and there are a few accredited institutions around the world that offer similar programs where students can receive official certifications for course completion. Thus, the experiences and lessons learned from the SFM-OER program can help to assess online forestry pedagogy, offer insights for improvements to the discipline, and help other institutions develop courses that address the needs of their own forestry faculties. This is especially relevant in looking at the rapid development of higher online forestry courses globally and regionally.

This study provides a comprehensive evaluation of the SFM-OER program in terms of student experiences through an assessment of course completion rate and learner’s feedback surveys. This study specifically aims to evaluate student demography, perceptions, attitudes, and levels of satisfaction with the online forestry courses and the adopted pedagogy from the SFM-OER, and to identify the program’s success and challenges. It also focus on students’ perceived effectiveness of learning activities within the program and their achievements, and the program’s ability to provide students with active and transformative learning experiences that improve their knowledge application and critical thinking skills. This study seeks to enhance learner-centered pedagogical development for online learning, propose ways forward for the SFM-OER program, and contribute to the advancement of higher forestry education.

The objectives of the study are to:

- Analyze the significant trends in demographics and characteristics of online learners in the SFM-OER program
- Evaluate students’ achievement and their perception and satisfaction with instructional design, learning technology, pedagogies, and learning activities in the SFM-OER online courses
- Identify the challenges and success of the online SFM-OER education program and discuss the program’s improvement

## Materials and methods

**Fig. 1:**
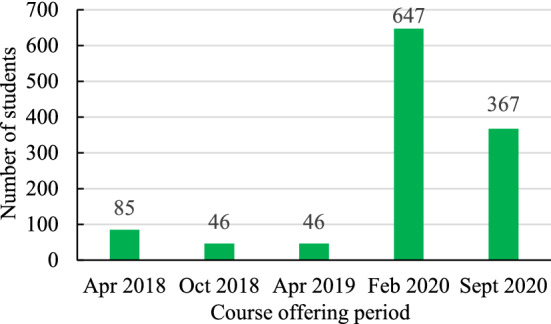
Number of survey respondents

For this analysis, 1,191 post-course student feedback surveys were collected over five course-offering sessions in April 2018 (85 respondents), October 2018 (46 respondents), April 2019 (46 respondents), February 2020 (647 respondents), and September 2020 (367 respondents) (Fig. 1). The courses offered between 2018 and 2019 consisted of one to two courses enrolled by 100 − 200 students in each cohort. Courses offered in February 2020 consists of seven courses whereas courses offered in September 2020 comprised of five courses. Only post-course survey data was used from Sustainable Forest Management in the Changing World (FODE 001), Forest Governance, Public Relations, and Community Development (FODE 002), International Forestry Issues, Institutions, and Multi-Lateral Agreements (FODE 003), and Restoration of Degraded Forest and Plantation Development and Forest Resource Management and Protection (FODE 005) OER courses for this study. It is comparable to the previous data in 2018 and 2019. These courses were similar to other regular university credit courses in which students typically spend 8 − 9 weeks to complete. While offering the course, a team-based approach, consisting of a course running manager, instruction and learning designer, lead instructor, and co-instructors including teaching assistants, was adopted to repurpose and deliver these OER courses. These open education resource courses were implemented using the Canvas Learning Management System (LMS) to support maximum instructor-student and student–student interactions. See https://apfecm.forestry.ubc.ca/sfm-online-courses/ for a detailed curriculum structure of the courses used in this research.

**Fig. 2:**
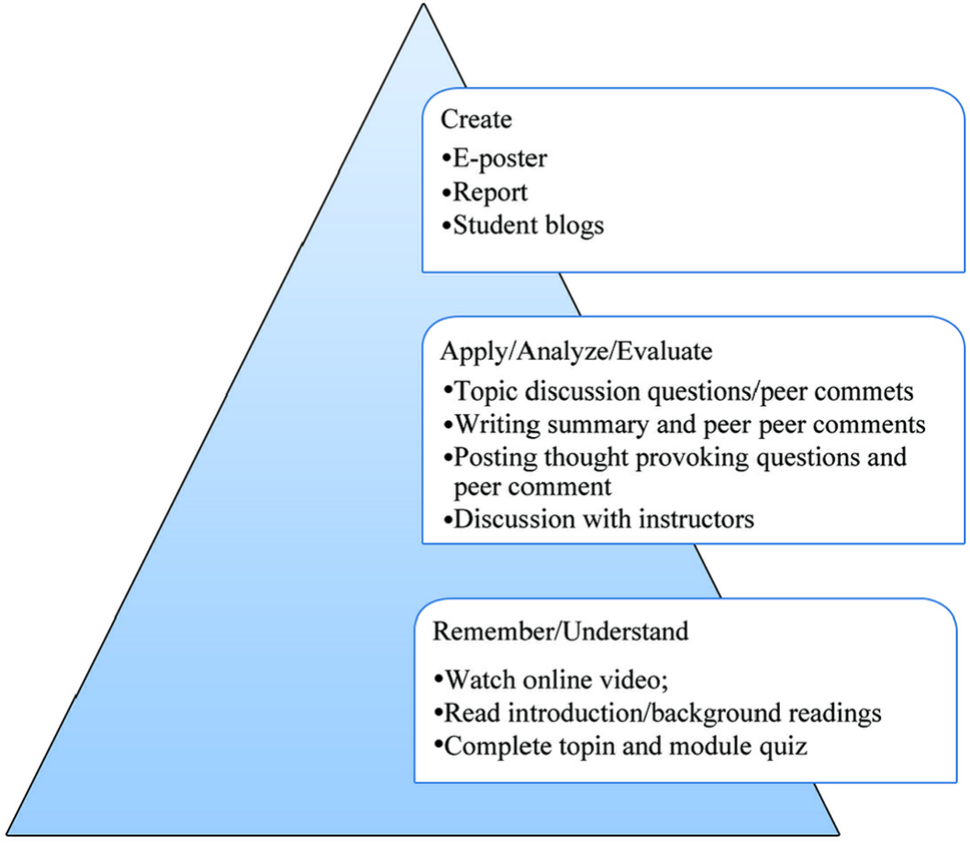
A typical example of learning-centered learning based on Bloom’s Taxonomy adopted in repurposed online learning

During teaching, our practices moved from lecture-based instruction to more learner-centered learning with student participation and peer interaction in discourse and co-creation of course materials. This was guided by Bloom’s Taxonomy of the hierarchical ordering of cognitive skills from less complexity to highest complexity form of learning (Fig. 2). This allowed students to engage with their peers and compare knowledge and experiences with their own, therefore gaining practical and applicable knowledge from other regions. With this approach, students learned about SFM, climate change, biodiversity conservation, forest restoration, and governance issues, and, most importantly, developed their critical analysis, writing, argumentation skills, and applied learned knowledge into practice to address various SFM issues in the region (Zeng et al. 2019).

During the course, students watch online videos and read learning materials to complete various mandatory graded assignments, (for example, weekly quizzes, response to discussion questions, reading peer-reviewed papers and writing summaries, peer comments, and writing a report), required to submit within a deadline. They also received feedback and encouragement from the instructor in their assignments for improvement and motivation. The passing grade for each course was set to a minimum of 60%. After completing each course, students were asked to complete the post-course survey prepared in Qualtrics embedded in Canvas LMS. There were 40 questions similar to all the five offerings. However, a few new questions were added later, particularly from 2019 (see the result section). Broadly, these questions were related to the demography of the students, such as their gender, fields of study, countries of origin, and information about the type of technology they were using to complete course requirements. They were also asked to use a Likert scale to communicate their experience and perceptions towards course workloads and assignment difficulty and provide feedback on how effective they found the learning activities, technology, and instructional design applied in the course. Students were given the opportunity to provide written reflections on challenges they faced and to make suggestions for improvements.

Broad outline and characteristics of questions in student feedback survey.

- Student demographics and characteristics
- Student accessibility to learning technologies
- Student time commitment and workload feedback on coursework
- Student perceived effectiveness of instructional mediums and activities
- Impacts on students’ perceived knowledge of subject matter and skills in critical thinking and expression

This data was first processed in Qualtrics, combining all the post-course survey data and descriptive analysis in MS EXCEL to produce informative graphs and tables. This analysis was then interpreted using existing literature regarding online education, higher forestry education, and online forestry education to inform how the SFM-OER program is achieving success in online higher forestry education and how it can be improved.

## Results

### Demography and motivation of students

The majority of students who participated in these repurposed online courses were represented by universities in China (63%), followed by Nepal (10%), Bangladesh (9%), Pakistan (4%), Myanmar (2%), India (1%), Indonesia (1%), Vietnam (1%), Japan (1%), Chile (1%) and other universities across South-east Asia, Central Asia, East Africa, and West Africa. Data collected from April 2019 to September 2020 revealed that the majority of students identified themselves as female (mean, hereafter Mean = 62.1%, standard deviation, hereafter SD = 2.9%) (Table 1), were predominately between 20 to 30 years of age (Mean = 78.7%, SD = 9.1%) (Table 1). From April 2018 to April 2019 over 50% of respondents were graduate-level students, but after this period, the demographic shifted to over 50% undergraduate students. Respondents who identified themselves as forestry instructors, forestry practitioners, forest enforcement officers, or others made up a small percentage of the students across all course offering periods (Table 1).

**Table 1:** Demography of students expressed as a percentage (%)

Demography	Apr 2018	Oct 2018	Apr 2019	Feb 2020	Sep 2020	Mean	SD
*Gender*							
Male*			34.8	38.2	40.6	37.9	2.9
Female*			65.2	61.8	59.4	62.1	2.9
*Age group*							
Under 20	0	13	8.7	21	14.2	14.2	4.4
20 − 30	93	67.4	78.3	77	77.9	75.2	4.5
30 − 40	5.9	15.2	13	1.2	6.3	8.3	5.5
Over 40	1.2	4.3	0	0.8	1.6	2.0	1.6
*Education level*							
Undergraduate	41.2	34.8	21.7	55.5	67.3	44.1	15.9
Graduate	51.8	52.2	73.9	39.7	24.8	48.5	16.2
Forestry instructor	1.2	2.2		1.1	1.6	1.52	0.4
Forestry practitioner	3.5	4.3	2.2	1.5	3.8	3	1.0
Forest enforcement officer	3.5	4.3	2.2	0	2.7	3.17	1.5

^*^This data was not collected in April 2018 and Oct 2018 offerings

**Fig. 3:**
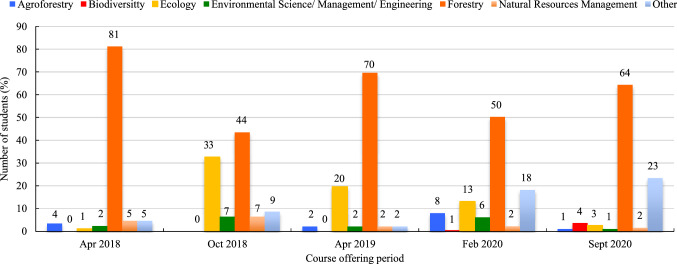
Student majors (expressed in %)

Most students who completed a course were forestry majors (Mean = 61.8%, SD = 15.1%) followed less closely by ecology majors (Mean = 13.8%, SD = 12.9%), although the number of students enrolled in ecology varied greatly between course offerings (Fig. 3). Most remaining students responded that they were enrolled in ‘other’ not listed disciplines (Mean = 11.4%, SD = 9.0%), especially after the Covid-19 pandemic began. The remaining disciplines that were represented in the program include agroforestry (Mean = 3.7%, SD = 3.0%), environmental science/management/engineering (Mean = 3.7%, SD = 2.5%), natural resources management (Mean = 3.5%, SD = 2.1%), and biodiversity (Mean = 0.9%, SD = 1.6%) (Fig. 3).

**Fig. 4:**
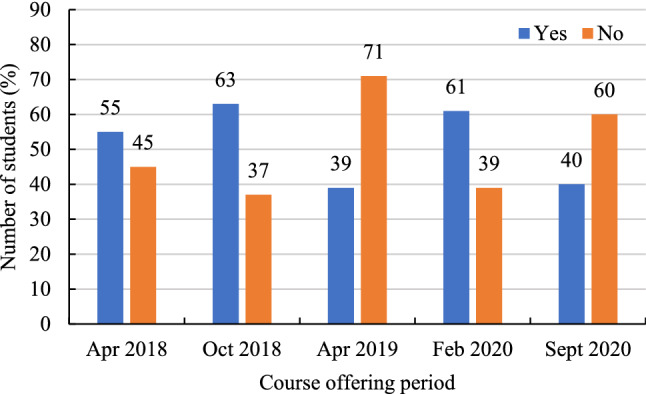
Students participating in an English learning environment for the first time (expressed in %)

Respondents between April 2018 and September 2020 were from more than 40 countries. The majority were located in China (63%), followed by Nepal (10%), and Bangladesh (9%). In April 2018, October 2018, and February 2020, 55.3% and 63.0% of learners participated in an English learning environment for the first time. In April 2019 and September 2020, this was reversed, where 60% of learners had previously experienced an English language learning environment (Fig. 4).

**Fig. 5:**
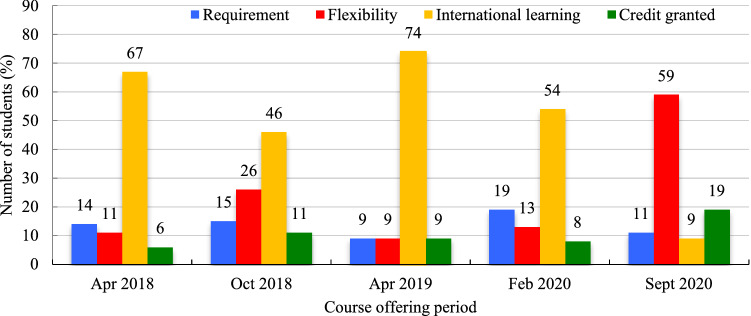
Students motivation for participation in the course (expressed in %)

The primary reason students enrolled in SFM-OER courses was to gain an international learning experience (Mean = 60.0%, SD = 10.9%), ranging between 46.0% in October 2018 to 74.0% in April 2019 offerings (Fig. 5). Other reasons for enrollment included the flexibility of online learning (Mean = 13.6%, SD = 7.1%), mandatory course requirements (Mean = 15.2%, SD = 4.2%), and to earn a course credit (Mean = 9.0%, SD = 2.1%); however, these other reasons for enrollment were not as popular as the international attraction of the program (Fig. 5).

###  Student accessibility to learning technologies

Laptop computers were used by an average 71.2% (SD = 6.5%) of learners to access course material (Table 2). A much smaller proportion used smartphones (Mean = 16.4%, SD = 7.2%), desktop computers (Mean = 11.8%, SD = 4.0%), and tablets (Mean = 4.9%, SD = 1.3%) (Table 2). Students were inclined towards the use of google chrome (Mean = 54.9%, SD = 14.3%) over other web browsers such as Internet Explorer (Mean = 18.9%, SD = 8.9%), Firefox (Mean = 9.7%, SD = 1.2%), and others (Mean = 16.5%, SD = 6.7%) (Table 2).

**Table 2:** Learning technologies used expressed as a percentage (%)

Learning technology	Apr 2018	Oct 2018	Apr 2019	Feb 2020	Sep 2020	Mean	SD
*Device used*							
Desktop	15.3	8.7	15.2	13.3	6.5	11.8	3.6
Laptop	62.4	76.1	78.3	72.0	67.0	71.2	5.8
Tablet		4.3	6.5	5.3	3.5	4.9	1.1
Smart Phone	22.4	10.9		9.4	22.9	16.4	6.3
*Browser*							
Internet Explorer	4.7	19.6	26.1	26.7	17.4	18.9	8.9
Firefox	8.2	10.9	10.9	9.9	8.7	9.7	1.2
Chrome	74.1	54.3	50.0	35.1	60.8	54.9	14.3
Other	12.9	15.2	13.0	28.3	13.1	16.5	6.7
*Internet access*							
University	43.5	63.0	87.0	10.5	36.5	48.1	28.7
Home	47.1	34.8	6.5	86.1	59.9	46.9	29.5
Public café	1.2	0.0	2.2	0.8	0.5	0.9	0.8
Other	8.2	2.2	4.3	2.6	3.0	4.1	2.4

The number of students accessing course material from home decreased from 47.1% in April 2018 to 6.5% in April 2019, and university access rose from 43.5% to 87.0% in this period (Table 2). In the February 2020 course offering, home access increased to 86.1%, and university access decreased to 10.5%. Home access remained the dominant location for accessing the course in September 2020 at 59.9%, but access from the university increased again to 36.5% (Table 2).

**Fig. 6:**
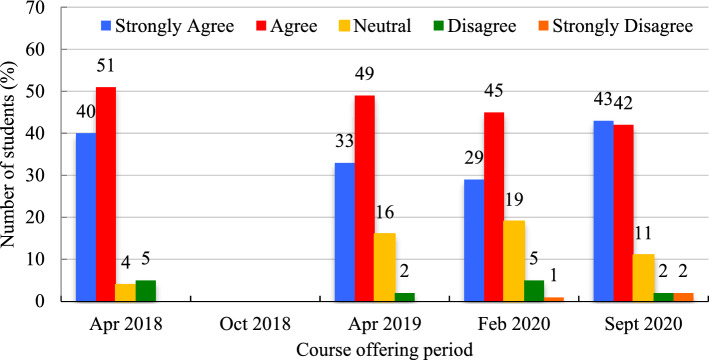
Course platform user-friendliness (expressed in %)

The majority of respondents in every course offering, except for the October 2018 term (for which there is no data), indicated their agreement regarding the user-friendliness and ease of use of the course platform (Fig. 5). ‘Agreement’ towards this statement remained between 42 and 51%, and ‘strong agreement’ between 29 and 42% (Fig. 6). ‘Neutral’ attitude towards this statement was highest (19%) in February 2020, when ‘strong agreement’ was lowest (29%). Students were asked in all five course offerings to indicate that 'they had no problems accessing the course to which they were largely in ‘agreement’ (Mean = 48.4%, SD = 4.7%) followed by ‘strong agreement’ (Mean = 29.2%, SD = 8.6%). A small number of students were ‘neutral’ (Mean = 14.8%, SD = 4.8%), ‘disagreed’ (Mean = 6.8%, SD = 3.6%), or ‘strongly disagreed’ (Mean = 1.7%, SD = 1.2%) towards this statement.

### Student time commitment and workload feedback on coursework

**Fig. 7:**
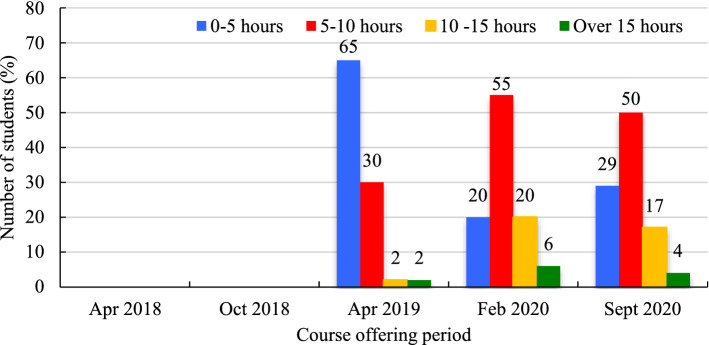
Average time spent on coursework per week

**Fig. 8:**
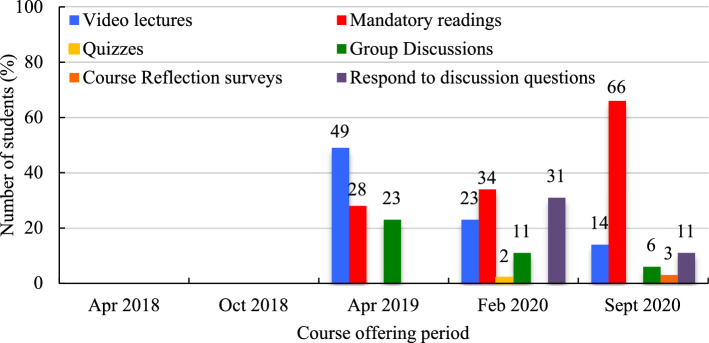
On which learning activities did you spend the most of your time?

Learners were surveyed about how much time they spent on coursework per week from April 2019 to September 2020. In April 2019, most learners (65%) typically spent up to 5 h working on course material (Fig. 7). This trend was changed in September 2019 and February 2020 when most students would spend 5 − 10 h of work per week on their course materials (50% and 55%, respectively). Students were also asked which learning activity they spent the most of their time on (Fig. 8). In April 2019, video lectures were reported as the most time spent activity (49%), followed by mandatory readings (28%) and group discussions (23%). In the February 2020 course offering, this changed to mandatory readings (34%), closely followed by responses to peer discussion questions (31%), and video lectures (23%). Finally, in September 2020, mandatory readings were predominately seen as the most time-consuming activity (66.0%). Quizzes and course reflections surveys were very seldom regarded as the most time-consuming activities, if at all.

Almost half of the respondents consistently ‘agreed’ that the course workloads were manageable (Mean = 49.2%, SD = 3.5%) (Table 3). The remaining students ‘strongly agreed’ (Mean = 23.6%, SD = 6.3%) or were neutral (Mean = 21.4%, SD = 8.2%). A small minority ‘disagreed’ that the course workload was manageable (Mean = 5.2%, SD = 1.9%) and an even smaller number ‘strongly disagreed’ (Mean = 1.7%, SD = 1.1%). Half of the respondents also ‘agreed’ (Mean = 50.4%, SD = 3.2%) with the statement that ‘the number of assignments and discussions was appropriate for me’ (Table 3). On average, 29.2% (SD = 6.2%) ‘strongly agreed’ with this statement, and 14.4% (SD = 6.4%) were ‘neutral’. The strongest levels of dissatisfaction occurred in October 2018 when 14% of respondents stated they ‘disagreed’ that the number of assignments and discussions were appropriate, and the same number were ‘neutral’ about this statement. In other years, the level of ‘disagreement’ was relatively low at zero to 6%, and ‘strong disagreement’ always remained below 1%. Over half of the respondents ‘agreed’ (Mean = 53.4%, SD = 7.1%) that ‘the level of the assignments as appropriate’ (Table 3). In order following this, they ‘strongly agreed’ (Mean = 24.6%, SD = 6.8%), were neutral (Mean = 17.0%, SD = 5.1%), ‘disagreed’ (Mean = 4.6%, SD = 4.3%), and ‘strongly disagreed’ (Mean = 1.0%, SD = 0.0%).

**Table 3:** Students time commitment and workload expressed in percentage (%)

Student time commitment & workload	Apr 2018	Oct 2018	Apr 2019	Feb 2020	Sep 2020	Mean	SD
*The course workload was manageable*							
Strongly agree	28.0	30.0	14.0	21.0	25.0	23.6	6.4
Agree	45.0	54.0	51.0	49.0	47.0	49.2	3.5
Neutral	23.0	8.0	30.0	25.0	21.0	21.4	8.2
Disagree	3.0	8.0	5.0	4.0	6.0	5.2	1.9
Strongly disagree	3.0	0.0	0.0	1.0	1.0	1.0	1.2
*Assignments and discussions number was appropriate*							
Strongly agree	38.0	27.0	21.0	29.0	31.0	29.2	6.2
Agree	50.0	46.0	53.0	54.0	49.0	50.4	3.2
Neutral	5.0	14.0	23.0	14.0	16.0	14.4	6.4
Disagree	6.0	14.0	2.0	2.0	3.0	5.4	5.1
Strongly disagree	1.0	0.0	0.0	1.0	1.0	0.6	0.6
*The level of assignments and discussions was appropriate*							
Strongly agree	31.0	30.0	14.0	23.0	25.0	24.6	6.8
Agree	59.0	41.0	56.0	56.0	55.0	53.4	7.1
Neutral	9.0	19.0	23.0	18.0	16.0	17.0	5.2
Disagree	1.0	11.0	7.0	2.0	2.0	4.6	4.3
Strongly disagree	0.0	0.0	0.0	1.0	1.0	0.4	0.6

Finally, students were asked if the course activities and interactions met their expectations. Data for this was collected in every course offering period except for October 2018. Over half of students ‘agreed’ (Mean = 52.8%, SD = 4.8%) that the course activities did meet expectations, an average of 30.3% (SD = 5.0%) ‘strongly agreed’, and an average of 15.5% (SD = 3.3%) were neutral.

###  Students’ perceived effectiveness of instructional mediums and activities

*Quality of multimedia*

**Fig. 9:**
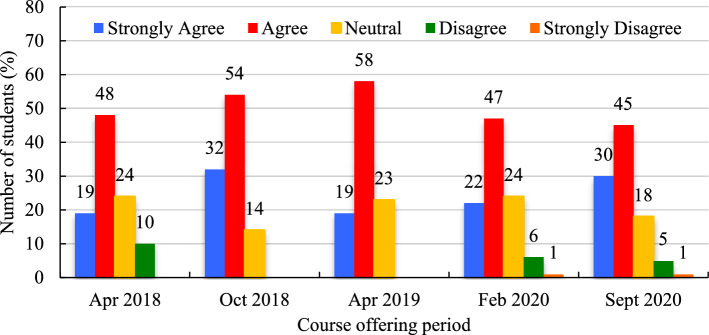
Quality of the multimedia used (expressed in %)

Students were provided with various instructional mediums and activities to help them learn the course content: video lectures, discussion forums, quizzes, and peer response writing activities. Throughout all five course offerings, students ‘strongly agreed’ (Mean = 24.4%, SD = 6.2%) or ‘agreed’ (Mean = 50.4%, SD = 5.4%) that the quality of the multimedia used was good (Fig. 9), ranging from 19% − 30% and 45% − 58% among different offerings. Levels of ‘disagreement’ and ‘strong disagreement’ were low (Mean = 7.0%, SD = 2.6% & Mean = 1.0%, SD = 0.0%). The number of students who were neutral about this statement were in the range of 14% to 24% (Mean = 20.6%, SD = 4.5%). Thus, most students appeared to be satisfied with the multimedia quality used during the course.

#### Effectiveness of reading activities

**Fig. 10:**
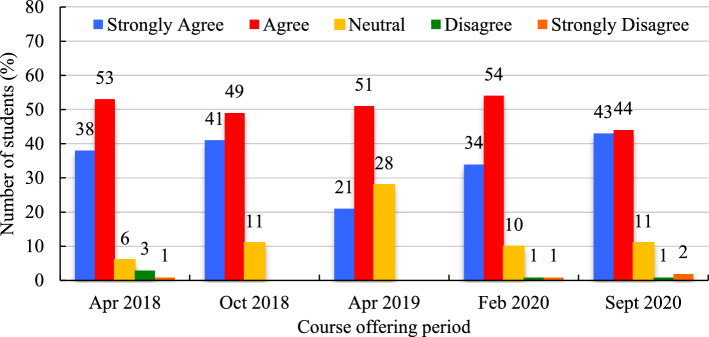
Reading activities effectively helped my learning in this course (expressed in %)

Student responses to ‘the readings effectively helped my learning in the course' indicated that, on average, most students ‘strongly agree’ (Mean = 35.4%, SD = 8.7%) or ‘agree’ (Mean = 50.2%, SD = 4.0%), with ranges from 34.0% − 43.0% and 44.0% − 54.0% respectively over the five offerings (Fig. 10). Very few students ‘strongly disagree’ (Mean = 1.3%, SD = 0.6%) or ‘disagree’ (Mean = 1.7%, SD = 1.2%). Generally, 6% to 11% of students were neutral about this statement, depending on the time of the course offering, except for the April 2019 semester, when 28% responded with ‘neutral’.

In the February 2020 and September 2020 periods, students were asked if the mandatory readings helped them to comprehend the readings and course content (Table 4). The majority either ‘strongly agreed’ (34% and 51%, respectively) or ‘agreed’ (60% and 43%, respectively). In these same two periods, students were asked if the 'key summary writings of readings' helped them comprehend the course readings and contents (Table 4). Again respondents ‘strongly agreed’ (42% and 50%, respectively) or ‘agreed’ (50% and 40%, respectively). The remaining students were neutral towards this statement for the two periods (10% and 9%, respectively), and a small percentage disagreed’ (1% and 1%, respectively) or ‘strongly disagreed’ (1% and 0%, respectively).

**Table 4:** Effectiveness of mandatory readings and key summary writings expressed as a percentage (%)

Learning activities	Feb 2020	Sept 2020	Mean	SD
*Mandatory readings*				
Strongly agree	34.0	51.0	42.5	12.0
Agree	60.0	43.0	51.5	12.0
Neutral	5.0	6.0	5.5	0.7
Disagree	2.0	0.0	1.0	1.4
Strongly disagree	0.0	0.0	0.0	0.0
*The key summary writings readings*				
Strongly agree	42.0	50.0	46.0	46.0
Agree	46.0	40.0	43.0	43.0
Neutral	10.0	9.0	9.5	9.5
Disagree	1.0	1.0	1.0	1.0
Strongly disagree	1.0		1.0	1.0

####  Effectiveness of video lectures

**Fig. 11:**
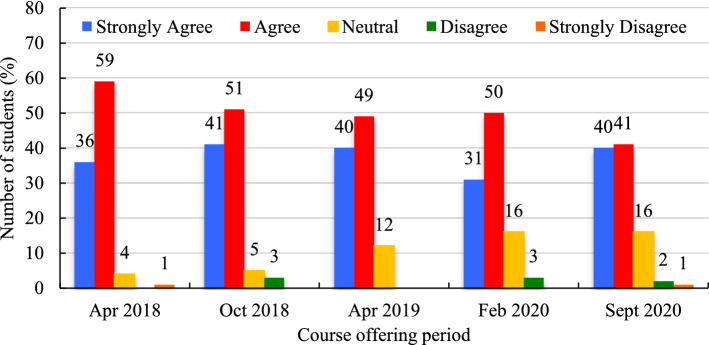
‘Video lectures effectively helped my learning in the course’ (expressed in %)

When presented with the statement ‘video lectures helped my learning in the course’ a combined average of 50.0% (SD = 6.4%) of the students ‘agreed’ (Fig. 11). Of the other half of the remaining students, 31.2% (SD = 4.2%) ‘strongly agreed’ with this statement while 10.6% (SD = 5.8%) were ‘neutral’, 2.7% (SD = 0.6%) ‘disagreed’, and 1% (SD = 0%) ‘strongly disagreed’. ‘Disagreement’ and ‘strong disagreement’ remained under 3% in any given period.

####  Effectiveness of quizzes

**Fig. 12:**
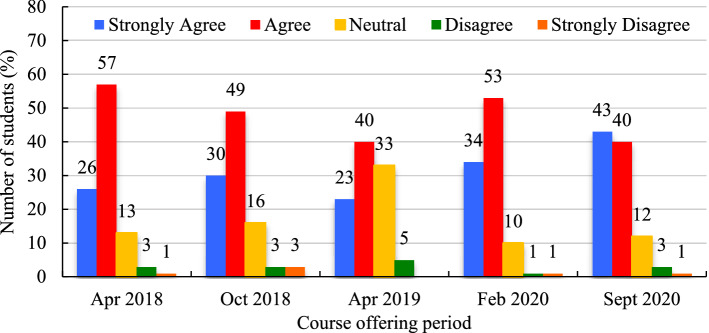
‘Self-test quiz effectively helped my learning in the course’ (expressed in %)

An average of 47.8% (SD = 7.7%) of students ‘agreed’ that self-test quizzes aided their learning in this course; 31.2% (SD = 7.8%) ‘strongly agreed’; 16.8% (SD = 9.3%) were ‘neutral’; 3.0% (SD = 1.4%) ‘disagreed’; and 1.5% (SD = 1.0%) ‘strongly disagreed’ (Fig. 12). Levels of ‘strong agreement’, ‘agreement’, and ‘neutrality’ fluctuated considerably, depending on the course period, with a range from 23% − 43%, 40% − 57% and 12% − 33%, respectively, for the two periods over the five course offerings but ‘disagreement’ and ‘strong disagreement’ with this statement remained below a collective 6% for any given course offering.

####  Effectiveness of discussion activities

The statement, ‘the online discussion effectively helped my learning in the course’, was presented to survey respondents between October 2018 and September 2020 (Table 5). Students ‘agreed’ with this statement (Mean = 48.0%, SD = 5.0%), ‘strongly agreed’ (Mean = 34.5%, SD = 6.6%), or were neutral (Mean = 15.5%, 5.7%). A small percentage ‘disagreed’ or ‘strongly disagreed’ with this statement (Mean = 1.8%, SD = 1.0% and Mean = 1.0%, SD = 0% respectively).

**Table 5:** Students perceived effectiveness of online discussion activities expressed as a percentage (%)

Effectiveness of online discussion	Apr 2018	Oct 2018	Apr 2019	Feb 2020	Sep 2020	Mean	SD
The discussion effectively helped my learning*							
Strongly agree		27.0	33.0	35.0	43.0	34.5	6.6
Agree		46.0	53.0	51.0	42.0	48.0	5.0
Neutral		24.0	12.0	12.0	14.0	15.5	5.7
Disagree		3.0	2.0	1.0	1.0	1.8	1.0
Strongly disagree				1.0	1.0	1.0	0.0
*The discussion questions were interesting and relevant*							
Strongly agree	46.0	24.0	16.0	33.0	41.0	32.0	9.5
Agree	44.0	59.0	65.0	52.0	43.0	52.6	8.3
Neutral	10.0	16.0	14.0	14.0	12.0	13.2	1.5
Disagree			5.0	1.0	2.0	2.7	1.7
Strongly disagree					1.0	1.0	0.0
*Group discussion helped me to connect with my peers*							
Strongly agree	39.0	35.0	26.0	41.0		35.3	6.7
Agree	48.0	43.0	56.0	35.0		45.5	8.8
Neutral	13.0	16.0	16.0	20.0		16.3	2.9
Disagree		3.0	2.0	4.0		3.0	1.0
Strongly disagree	1.0	1.0				1.0	0.0

* This data was not collected in April 2018 offerings

On average, 52.6% (SD = 8.3%) of students ‘agreed’ with the statement that ‘the discussion questions were interesting, relevant, and enhanced my learning’ followed by 32.0% (SD = 9.5%) who ‘strongly agreed’ and 13.2% (SD = 1.5%) who expressed neutrality (Table 5).

Almost half of the respondents were in ‘agreement’ (Mean = 45.5%, SD = 8.8%) that ‘group discussion helped me to connect with my peers’, followed by ‘strong agreement’ (Mean = 35.3%, SD = 6.7%), and ‘neutral’ (Mean = 16.3%, SD = 2.9%) for April 2018 to February 2020 (Table 5). ‘Agreement’ was always the most common response except in February 2020 when 'strong agreement' was rated higher.

#### Effectiveness of peer response activities

**Fig. 13:**
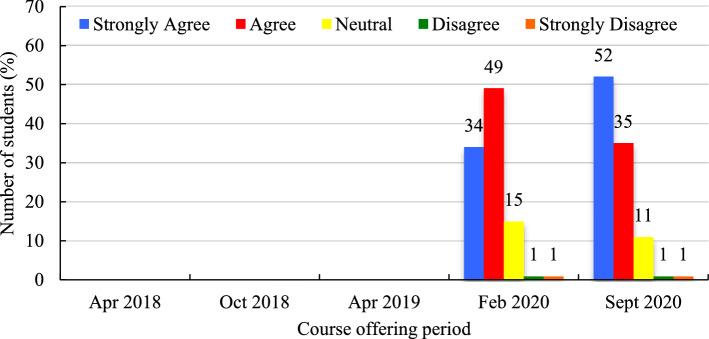
‘The peer response writing activity really helped me to reflect on course material’ (these data were not collected in April 2018, Oct 2018 and April 2019 offerings)

The statement ‘the peer response writing activity really helped me to reflect on course material’ introduced in the February 2020 and September 2020 course offerings revealed that respondents were mostly in ‘agreement’ (49% and 35%, respectively) and ‘strong agreement’ (34% and 52%, respectively), and a small handful were neutral (15% and 11%, respectively) (Fig. 13).

####  Preferences in learning activities

**Fig. 14:**
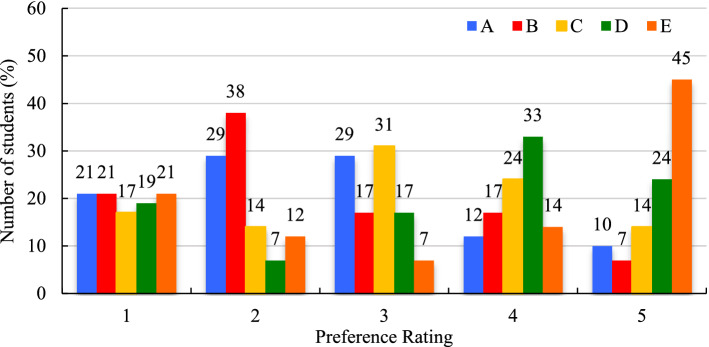
Course activity preferences April 2019 (expressed in %)

**Fig. 15:**
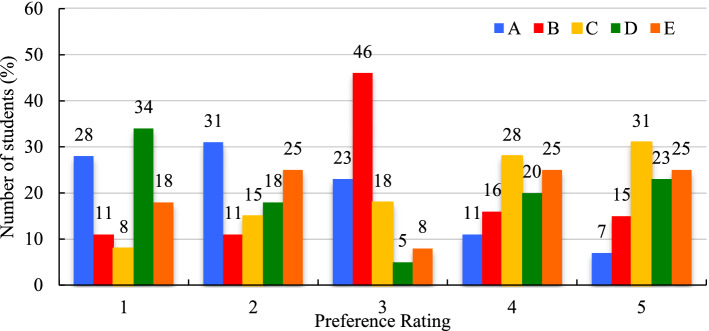
Course activity preferences February 2020 (expressed in %)

Learners in the April 2019 and the February 2020 course periods were asked to rank 5 different discussion-based learning activities (Table 6) from ‘most preferred’ to ‘least preferred’ using a scale of 1 to 5 (Figs. 14 and 15).

**Table 6:** List of learning activities

**A**	Watch online lecture videos and answer the discussion questions illustrating concepts/ principles/applications with examples from your own experience, or from your country region
**B**	Watch video case studies and answer the discussion questions providing solutions for identified constraints in the cases
**C**	Watch video case studies and answer the discussion questions explaining principles applied in the cases
**D**	Role play as a forest manager and answer the discussion questions addressing issues in your local/ region/ country
**E**	Read peer reviewed papers and answer the discussion questions tackling sustainable development targets in your country

Activity preferences were highly varied within and between the two course offerings. Rarely did more than a third of respondents assign any given activity the same ranking, except for activity **E** as the least preferred activity in April 2019 at 45%, and activity **B** as the third most preferred activity in February 2020 at 46% (Figs. 14 and 15). A weighted average ranking calculation in Excel revealed the order of preference in April 2019 to be **B**, **A**, **C**, **D**, and **E**, and in February 2020 it was **A**, **D**, **E**, **B**, and **C**.

###  Student achievements and their perceived impact on their knowledge of subject matter and skills in critical thinking and expression

**Fig. 16:**
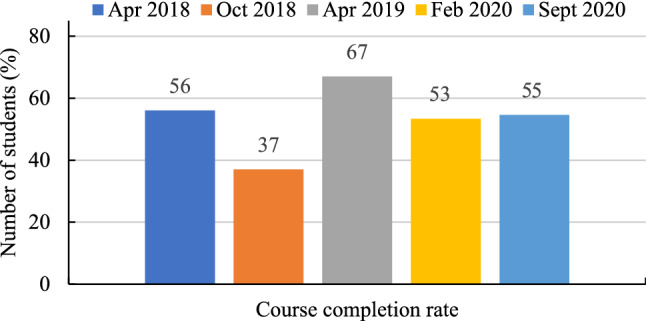
Course completion rate of students (expressed in %)

The average course completion rate over five offerings was 53%, with the highest in 2019 (67%) and the lowest (37%) in the 2018 October session. Completion rate for 2018 April 2020 Feb and 2020 September sessions were 56.0%, 53.4% and 54.6% respectively (Fig. 16).

Prior to beginning the program, most students regarded themselves as ‘new’ to the course content (Mean = 34.0%, SD = 17.0%) or only ‘somewhat familiar’ with it (Mean = 49.4%, SD = 10.8%); it was rare for students to feel ‘very familiar’ (Mean = 3.6%, SD = 3.2%) (Table 7). Respondents did not answer ‘familiar’ in the 2018 course offerings, but in succeeding courses, 12% to 29% of respondents felt that they were. Comparing these responses with the level of familiarity that learners reported having before taking a course indicates that those 'familiar' with the course material increased by 71%, 64%, 41%, 51%, and 9%, respectively, over the course periods (Table 7). Likewise, those who were ‘very familiar’ with the course material increased by 16%, 23%, 20%, 12%, and 40%, respectively (Table 7), indicating that student familiarity with the course increased substantially.

**Table 7:** Students’ perceived familiarity before and after taking course expressed as a percentage (%)

Level of familiarity	Apr 2018	Oct 2018	Apr 2019	Feb 2020	Sep 2020	Mean	SD
*Before taking the course*							
New	33.0	63.0	28.0	27.0	19.0	34.0	17.0
Somewhat familiar	58.0	33.0	46.0	60.0	50.0	49.4	10.8
Familiar			24.0	12.0	29.0	21.7	8.7
Very familiar	9.0	4.0	2.0	1.0	2.0	3.6	3.2
*After taking the course*							
New	2.0	3.0	7.0	4.0	4.0	4.0	1.9
Somewhat familiar	2.0	6.0	7.0	21.0	16.0	10.4	7.8
Familiar	71.0	64.0	65.0	63.0	38.0	60.2	13.0
Very familiar	25.0	27.0	22.0	13.0	42.0	25.8	10.5

**Fig. 17:**
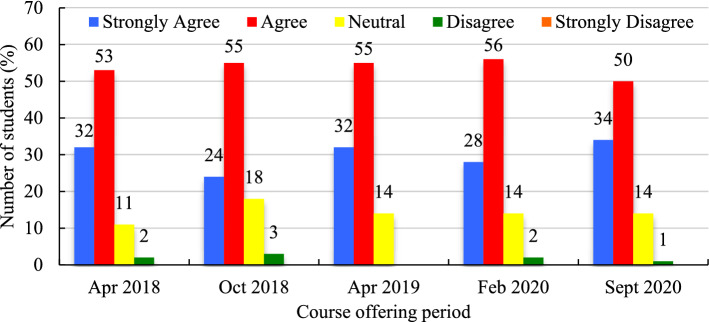
The course helped to advance my knowledge on the subject matter’ (expressed in %)

More than half of all students in each of the five courses ‘agreed’ (Mean = 53.8%, SD = 2.4%) that the courses ‘helped to advance their knowledge on the subject matter' with the highest 56% in Feb 2020 offerings (Fig. 17). Additionally, an average of 30% (SD = 4%) ‘strongly agreed’ with this statement ranges from 24% − 34% over five offerings, 14.2% (SD = 2.5%) were neutral, and 2% ‘disagreed’ (SD = 0.8%).

In February 2020 and September 2020, students were asked if participating in the course improved their ‘critical analytical thinking’, ‘improved argumentation and discussion skills’, and ‘improved their English writing skills and writing summaries’ (Table 8). More than 85% of students in each course period responded that they ‘agreed’ or ‘strongly agreed’ to each statement; however, the majority tended to ‘agree’ rather than ‘strongly agree’. Moreover, between 11 and 12% were ‘neutral’ towards each statement in each course offering, and a total of less than 3% ‘disagreed’ or ‘strongly disagreed’.

**Table 8:** Perceived improvement in student critical analysis, argumentation, and writing skills expressed as a percentage (%)

Improvement in students’ skills	Apr 2018	Oct 2018	Apr 2019	Feb 2020	Sep 2020	Mean	SD
Improve critical analytical thinking*							
Strongly agree				33.0	40.0	36.5	5.0
Agree				52.0	45.0	48.5	5.0
Neutral				12.0	13.0	12.5	0.7
Disagree				2.0	2.0	2.0	0.0
Strongly disagree				1.0	1.0	1.0	0.0
Improve argumentation and discussion skills*							
Strongly agree				37.0	42.0	39.5	3.5
Agree				50.0	46.0	48.0	2.8
Neutral				12.0	11.0	11.5	0.7
Disagree				1.0	1.0	1.0	0.0
Strongly disagree							
Improve English writing skills and writing summary*							
Strongly agree				36.0	39.0	37.5	2.1
Agree				50.0	47.0	48.5	2.1
Neutral				12.0	13.0	12.5	0.7
Disagree				2.0	1.0	1.5	0.7
Strongly disagree				2.0	1.0	1.5	0.7
The course met expectations							
Strongly agree	33.0	26.0	27.0	29.0	36.0	32.5	5.0
Agree	52.0	42.0	57.0	53.0	51.0	52.0	1.4
Neutral	12.0	26.0	16.0	15.0	11.0	13.0	2.8
Disagree	2.0	5.0		2.0	2.0	2.0	0.0
Strongly disagree				1.0	1.0	1.0	0.0

*These data were not collected in April 2018, Oct 2018 and April 2019 offerings

Finally, when students were asked if the courses met their expectations, the majority ‘agreed’ (Mean = 51%, SD = 5.5%) or ‘strongly agreed’ (Mean = 30.2%, SD = 4.2%) (Table 8). An average of 16% (SD = 6.0%) of students were ‘neutral’, 2.8% (SD = 1.5%) ‘disagreed’, and 1% ‘strongly disagreed’.

###  Challenges and feedback reported by students

Students were asked to identify challenges and provide feedback (reported in the form of statements) while participating in the online course. The majority of students reported a positive learning experience; a few also reported challenges such as heavy workloads and tight schedules, problems with network access, a desire for more direct interactions with instructors (synchronous online learning), language communication barriers, and difficulties with different time zones which made assignment deadlines confusing.

Students also made suggestions for improvements. For instance, they suggested having video discussion forums and having a facilitator available to help provide direction, extending assignment deadlines, using social media to promote interactions with instructors and fellow students, adding subtitles for videos or providing the ability to download video content, and showcasing the best answers so that students have a template of what a well-done assignment should look like. Students also recommended including forestry courses such as agroforestry, forest hydrology and watershed management, biodiversity conservation, and wildlife management courses. Finally, respondents suggested that a final research paper or essay would be a welcome addition to a course.

## Discussion

###  Demographics

A higher proportion of female students completed courses between 2018 and 2020 than males, although forestry has been traditionally male-dominated (Macinnis-Ng and Zhao 2022). This observation is consistent with the recent FAO Asia Pacific forestry education report (Saengcharncha et al. 2021). Women in forest management are thought to emphasize forest preservation and management strategies that center around the environment and humans (Nordlund and Westin 2010). Thus, as a program that aims to teach learners about SFM for both people and the environment, the SFM-OER program seems attractive to the interests of female students by providing equal learning opportunities. It has also been found that many female graduate students have entered the discipline of forestry without prior intentions of doing so, and that they often come from related disciplines after learning more about forestry and how it may potentially suit their interests (Larasatie et al. 2020). This suggests that the SFM-OER program may be significant in motivating women to the discipline of forestry by offering courses that are easily accessible to a broad range of undergraduate female students regardless of their program of study.

In April 2018 to April 2019, students enrolled in the program were primarily graduates in the 20- to 30-year-old and the 30- to 40-year-old age brackets. This is in accordance with other studies that reported that online learners' demographic profile is often found to be older than the typical university age student (18 to 24-year-old) (House-Peters et al. 2019). The accessibility and flexibility of online courses are attractive to mature students who often have duties and responsibilities in their lives outside of academia. In February 2020 and September 2020, the outbreak of Covid-19 forced students to enroll in online education options, and this is likely the reason that the predominant education level switched from graduate to undergraduate during this time, as well as the reason for the increase in the number of students under 20 years of age.

###  Motivation to participate in the course

Students were primarily attracted to the SFM-OER program for the international learning experience it provides. The borderless qualities of OER courses allow learners to network with professors and peers worldwide. Furthermore, students can experience methods of instruction, course materials, concepts, and case studies that are different from what they are exposed to at their local institutions. Thus, students can explore aspects of studying abroad without leaving their home countries and learn foreign forest management techniques that may prove useful in future academic settings or workplaces. They can then apply these tools and combine them with techniques from their own regions to develop creative solutions to complex problems.

The opportunity to study the English language is another potential benefit of an international learning experience. Proficiency in English is an essential skill for global employment opportunities, and these online learning environments provide an ideal place for learners to practice their English language skills through written interactions with peers and professors. To reflect this, learners indicated that they felt their English writing and summary skills improved. Online written correspondence also removes the social pressure in face-to-face classrooms where students might feel uncomfortable having to quickly verbalize their thoughts in their native languages (Arasaratnam-Smith and Northcote 2017).

### Learning activities

Effective forestry education models are interactive, hands-on, and allow students innovative learning opportunities by providing access to various learning activities. The SFM-OER program offers diverse learning activities, including discussions and debates, role-playing, self-reflection, and problem-based and case-based learning approaches that help students develop critical thinking skills. Furthermore, students can engage with course content through various mediums, including video lectures, reading materials, discussion forums, quizzes, and peer response writing activities, signifying the importance of using diverse learning activities to satisfy the needs of various individual learning styles that helps diverse learners best understand, remember, and interpret information. It has been found that online learning is improved when courses offer a variety of learning activities because students can access modes of instruction that complement their personal learning styles but will also be challenged and made more versatile students by learning activities that they do not naturally favor (Zapalska and Brozik 2006). The lack of clear preferences observed amongst students when they were asked to rank their favorite course learning activities is likely reflective of a student body with diverse learning styles within each course offering. Changes in learner demographics may also have impacted the results between the April 2019 course offering (predominately consisting of graduate students in forestry programs who have previously studied in English) and the February 2020 course offering (primarily first-time English learners who were completing undergraduate degrees in a variety of disciplines). The preferred learning activities may have complemented each demographic groups’ strengths, interests, learning styles, and/or knowledge gaps in ways they felt were beneficial to their learning. These diverse learning activities and mediums, especially peer discussion activities, promote active learning — an instructional method that engages students in the learning process and encourages them to reflect upon and be critical of what they are learning. Active learning techniques have been shown to promote deeper levels of understanding, knowledge retention, creative problem-solving, improve soft skills, and increase employability (Arnold et al. 2019). The survey result indicates a high degree of student satisfaction regarding the instructional design, learning activities, and pedagogical practices applied within the SFM-OER program.

Learning activities adopted in SFM-OER offerings are designed to achieve all six objectives of Bloom’s taxonomy so that they will retain and comprehend information, develop critical analytical thinking skills, apply what they have learned to new scenarios, and develop new ideas (Fig. 2; Bloom et al. 1956). It is conceivable that objectives 1 and 2 of Bloom’s Taxonomy have been achieved in the SFM-OER course design. Responses to the feedback surveys reveal that learners’ perceived familiarity with the course material improved before and after taking a course, that they felt their knowledge and levels of understanding of the subject increased by taking these courses, that the video content, reading materials, and discussion activities helped with their learning and enhanced their knowledge, and that their comprehension of course content was improved by writing key summaries of readings. The majority of respondents agreed that taking SFM-OER courses and the discussion activities, specifically improved their critical analytical thinking skills and their augmentation and discussion skills, indicating that all of Bloom’s taxonomy up to number 5 are being met subjectively. Other assessments of the SFM-OER program have explored how discussion activities and peer response writing activities help meet all six objectives of Bloom’s Taxonomy by providing students with opportunities to discuss core ideas with peers, essential for in-depth exploration of their new knowledge and helps to build connections between real-world issues and subjects in the classroom (Zeng et al. 2020).

###  Technology accessibility

The course platform, Canvas, was considered user-friendly and easy to use by survey respondents and they ‘agreed’ or ‘strongly agreed’ that they had no problems accessing the course. Each course webpage followed a standard format, creating a sense of unity and making them easy to navigate for students taking multiple courses. Students were also 20- to 30-year-old and their positive responses may reflect the technological awareness of the Millennial and Zoomer generations who began computer-based learning at an early age. Moreover, most students preferred accessing the course via google chrome on their laptops, indicating that Canvas and the media content provided for these courses operate well with these commonly used technologies. Findings of this study suggest that using user-friendly learning management systems such as ours is crucial to enhancing student learning experiences, access to learning materials, enhance interaction between instructor and learners, and peer to peer, including timely support (Roddy et al. 2017). Hence, universities need to adapt their online education systems to meet changing education needs and trends (Saengcharnchai et al. 2021).

### Workloads and time management

There were three course offering periods where learners were asked about their time on coursework and specific course activities. Students in April 2019 reported video lectures as the most time-consuming activity and that they spent between up to 5 h on coursework per week. However, students in February 2020 and September 2020 felt reading activities were the most time-consuming and tended to spend 5 to 10 h on coursework per week. These changes may be a result of shifting demographics between course offerings. As previously mentioned, the April 2019 term primarily consisted of forestry graduate students, while in February and September 2020, learners were enrolled in many disciplines at the undergraduate level. Higher-level students are more experienced at reviewing and interpreting academic literature, so completing reading assignments is likely a less time-consuming process for them than for undergraduate students. On the other hand, watching video lectures takes a predetermined amount of time and is an activity that is consumed at an equal rate amongst all levels of experience.

Moreover, student responses to the online transition during the initial phase of the pandemic reveal that they lacked self-discipline and struggled with focus (Bao 2020). Reading activities require higher levels of concentration than videos; thus, they may have felt more challenging and time-consuming during this time. Overall, in every course offering, learners agreed or strongly agreed that course workloads, the number of assignments and discussions, and their level of difficulty were manageable and appropriate, suggesting that they were satisfied with course workloads and the required time commitments regardless of differences in opinions on what the most time-consuming activities were between course offering periods.

###  Main challenges experienced in the online setting

Forestry has been traditionally taught as a hands-on discipline and field visits to forest management sites are a regular occurrence. The inability of online courses to provide hands-on skills and develop well-connected cohorts of students has been noted as a possible drawback to higher forestry education online (Dodson and Blinn 2022). The loss of field sessions may not be as critical in the SFM-OER program, where the focus is on broadscale multidisciplinary approaches to forest management rather than on regional silviculture or forest operations. In this case, the trade-off between hands-on learning and online learning is the opportunity for students to explore how SFM is being addressed from local to global scales. Another commonly cited challenge with online learning is that it is difficult to replicate face-to-face interactions and relationships in a classroom setting (Arasaratnam-Smith and Northcote 2017; Dodson and Blinn 2022). Furthermore, studies that examined the transition to online learning after Covid-19 found that feelings of isolation from their peers have been linked to poorer academic performance amongst learners (Paudel 2021). This is likely true for learners in the SFM-OER program who mostly worked from their university campuses before 2020 and at home after the Covid-19 outbreak. The resulting isolation and lack of connection between students and instructors is a significant consideration for administrators of the SFM-OER program if they wish to foster global connections between learners and instructors.

## Way forward

### Potential applications of the OER in the post-Covid-19 era

As new and urgent issues emerge in the field of forestry due to global changes in the environment and society, the characteristics of the SFM-OER program are a flexible model for providing up-to-date, multilateral forestry education accessible to diverse learners (Kanowski 2020). Individuals, instructors, institutions, and others may continue to modify how these courses are used to ensure that future forest managers receive the multidisciplinary and broad set of skills and knowledge required for them to succeed. For instance, instructors in hybrid learning environments may assign modules from one or several OER courses to their students to expose them to various SFM concepts they can discuss later in class.

The Covid-19 pandemic revealed unexpected value for the SFM-OER program as an emergency forestry education alternative during global school closures. This event proved the program's strength, which maintained its particularly optimistic pre-Covid feedback survey results throughout the February 2020 and September 2020 course offerings, despite reports of decreased focus and struggles with self-discipline amongst learners during this time elsewhere (Bao 2020). As a result, many students and instructors actively explored online higher forestry education, and there will likely be a growing demand for more high-quality online forestry education courses in the future. Thus, managers of the SFM-OER program could reasonably add several new courses in response to this, and this will provide learners with a broader pool of knowledge to grow the multidisciplinary understanding they will need to enter the forestry-related workforce.

APFNet and partners created the SFM-OER program to be a training opportunity for practicing foresters to advance their existing skills and professional careers. However, this analysis has shown that the demographic breakdown of learners enrolling in instructor-led offerings of this program mainly consisted of undergraduate or graduate-level students. If the managers of the SFM-OER program wish to expand their learner base, they should explore ways to advertise the program to practicing foresters and a broader subset of forestry-related institutions outside of the academic world. For instance, they might explore the benefits of a partnership with a forest industry representative or opportunities to create a training program specifically aimed at these individuals.

###  Instructional models in the post-Covid-19 era

Interest in studying forestry via online is likely to increase, as learners become more accustomed to distance learning environments, as technology improves, and as future disasters and pandemics induced by climate change and biodiversity loss threaten to disrupt the accessibility of face-to-face classrooms. Instructional models will likely adapt to these circumstances by offering new and innovative ways for learners to learn and explore course content and interact with their instructors and peers. Hence, the emerging instructional models for using SFM-OER effectively in the post-Covid-19 era may simultaneously deliver course content and activities to both in-person and remote groups and can be fully asynchronous delivery or a combination of asynchronous and synchronous sessions. For instance, in a concurrent hybrid model, in-person and remote students can attend class synchronously. In the asynchronous hybrid model, instruction in an in-person setting can be recorded and made available to remote students to view. In the sequential hybrid model, in-person and remote students can meet in separate, consecutive sessions where instruction can be repeated. In a multi-section hybrid mode, specific sections can be dedicated to either in-person or remote groups by different instructors. In an alternating hybrid model, students can be rotated to required in-person instruction or activities. This will benefit from creating a globally connected community of learners who can exchange ideas across international boundaries. Furthermore, as the technology used to deliver online forestry education improves, a greater variety of learning activities can be offered to students to meet their personal learning styles and to best provide the educational outcomes that course creators are attempting to deliver.

###  Recommendations

A major draw of the SFM-OER program is the international learning experience it offers; thus, fostering and enhancing this aspect of the OER courses should be a priority going forward. Connecting students and encouraging them to exchange ideas regarding how to address wicked problems of SFM, including the development of new forestry online courses currently lacking in our OER as suggested by students, is a necessary way forward. Well-designed discussion activities have been shown to help build relationships between online learners (Arasaratnam-Smith and Northcote 2017) and to exemplify this, learners who completed SFM-OER courses tended to ‘agree’ or ‘strongly agree’ that they enjoyed working with their discussion groups and felt that these activities helped to connect them with peers. Moreover, evidence has suggested that learners who remain connected to online learning technologies throughout the day develop a stronger sense of community than those who log in at pre-scheduled times (Arasaratnam-Smith and Northcote 2017). Instructors might hold online office hours, or periodically divide their classes into smaller break-out sessions so that students may have discussions about course topics in groups that are less intimidating in size and accessible for more people to share ideas. Instructors may also want to encourage students to build friendships and communities outside of formal instruction on social media or in person when possible (Dodson and Blinn 2022). These relationship-building activities should begin as soon as possible, and instructors may want to launch them using creative and fun icebreaker activities through discussion forums or other communication platforms.

Lee (2021) stresses that the openness of online courses and technological innovation can exist at odds with one another and might exclude minorities. As technology improves the ability to provide more innovative teaching materials, instructors should be aware of how this might exclude minority students without a certain ability to access the content. For instance, some economically disadvantaged learners may not have flexible schedules or be unable to afford the high internet speeds required to participate in activities such as real-time video discussions over Zoom. Supplying students with recordings of video lectures, PDF copies of lecture PowerPoints, subtitles on video content, opportunities to form peer support groups, and a high degree of diversity in the learning activities can assist with maintaining the openness of online learning in the SFM-OER program while also allowing for innovation. These resources will also benefit those studying English for the first time who can use multiple sources to help them interpret and understand the course material.

Students ‘agreed’ or ‘strongly agreed’ that the difficulty of assignments and workloads was appropriate, but in written feedback, some noted that they had issues due to their tight schedules and heavy course workloads outside of the SFM-OER program. They also indicated that they sometimes felt confused regarding assignment deadlines due to time zone differences. This suggests that learners may require assistance with time management. For instance, a precise calendar schedule layout, greater flexibility in submission deadlines, or stronger encouragement for students to reach out to their instructors if they are experiencing confusion or difficulty completing assignments in due time. Instructors should also be encouraged to be aware of student learning capacities, especially in times of stress such as during the Covid-19 pandemic.

###  Limitation of the study and future research directions

This study demonstrated the challenges and successes of the SFM-OER program, mainly based on the subjective data from student feedback surveys. The only objective data collected were course completion rate. The study’s dependence on subjective data can be biased with positive responses, hence gathering, and analyzing more objective information on student achievement is needed to address this issue. More objective data can be obtained through assessment of learning in each course and through the learning analytics data in the learning management system about student behavior. It will be informative to compare the subjective results with objectively-measured student learning gains. Future evaluations may also wish to perform analysis that contributes to our understanding of the complex processes and mechanisms involved in effective online teaching and learning. For instance, regression analyses could reveal a significant understanding of demographic characteristics and various opinions on instructional design, learning preferences, and educational outcomes. More data collected over time regarding the most preferred learning activities may also reveal more robust patterns in student preferences, contributing to our understanding of an effective and efficient pedagogical model in online settings. The SFM-OER program has attracted a higher number of self-identified female over male learners, an unusual occurrence in forestry education. A more in-depth examination of this occurrence may provide meaningful insights into the factors that make the SFM-OER program especially appealing to female learners; this can then be applied to the larger question of making forestry education more attractive to women and other disadvantaged learners for the democratization of teaching and learning in forestry.

## Conclusion

This study provides insights into learner demographics, accessibility of technology, and the course platform's convenience in recent years, including the Covid-19 period. It further evaluates student achievements, their perceived success of the pedagogical approach and instructional design, and the effectiveness of the learning activities in promoting active and transformative learning with critical thinking and knowledge application. In the short period since its inception, the SFM-OER program has been successful, measured through students' perception, in its quest to provide OER in SFM to various learners. As the global state of forests remains dynamic over space and time, higher forestry education programs must build capacity of future graduates with a wide knowledge base and skill sets that allow them to manage forests as complex and adaptive systems. The SFM-OER program satisfies these demands by offering diverse and multidisciplinary courses that are freely accessible for a wide variety of learners. This can be attributed to team-based and student-centered learning approaches, while designing and delivering OER courses that promote student interaction, enhancing learning from their peers about how complex forest-related issues are being addressed in different regions around the globe. This study shows that these practices produce students satisfied with their SFM-OER course experiences, who feel competent in understanding major forestry issues, critically analyzing new information, and applying their newfound knowledge to situations outside the classroom. This analysis contributes to the further development of student-centered pedagogical development for online learning and provides insight into the ways forward for online higher forestry education while repurposing the existing OER courses in the post-Covid-19 era.
